# Supplementary material for: Tissue-specific and functional loci analysis of CASP14 gene in the sheep horn
Source: PLoS One. 2024 Nov 21;19(11):e0310464. doi: 10.1371/journal.pone.0310464 (PMC11581258; doi:10.1371/journal.pone.0310464)
Supplement: Attachment — Submitted filename: Response to Reviewers.docx [file pone.0310464.s004.docx]

Response to Reviewers

Dear Dr. Raza,

Thank you for giving us the opportunity to submit a revised draft of the manuscript “Tissue-specific and functional loci analysis of *CASP14* gene in the sheep horn” for publication in the *PLOS ONE.* We appreciate the time and effort that you and the reviewers dedicated to providing feedback on our manuscript and are grateful for the insightful comments on and valuable improvements to our paper. We have addressed the majority of the suggestions from both the reviewers and the editor, with changes highlighted in the revised manuscript. Please see below, in blue, for a **point-by-point** response to the reviewers’ comments and concerns. All page numbers refer to the revised manuscript file with tracked changes.

Reviewers’ Comments to the Authors:

**Reviewer #1:**

1. The language of the manuscript is very confusing. For example it is not clear whether the authors performed an RNA sequencing experiment on 15 sheep samples for thus study or they just retrieved data from some previously published study. The latter seems to be the case.

Author response: Thank you for pointing this out. We apologize for not clearly stating the data, and we have added a section on line 69 of the manuscript to explain our data processing.

Line 69: We performed RNA-Seq on soft horn samples from the 15 collected sheep.

2. Their numbers seems to be incorrect, on line 68 they talk about 2915 RNA-seq datasets. The authors need to double check this.

Author response: Thank you for catching this. Our RNA-Seq data consists of two main parts: one part is the RNA-Seq data from the 15 sheep generated in our lab, as mentioned earlier, and the other part includes 2915 collected RNA-Seq datasets from the National Center for Biotechnology Information (NCBI) database (https://www.ncbi.nlm.nih.gov/) and EBI (https://www.ebi.ac.uk/), which are publicly available. These two parts were combined for subsequent analysis. We have improved the description of datasets in lines 70-73.

Lines 70-73: A total of 2,915 high-quality RNA-Seq datasets were accessed from the National Center for Biotechnology Information (NCBI) and the European Bioinformatics Institute (EBI), and combined with RNA-Seq data of our 15 sheep for the following procedures.

3. The result section is very unclear and needs to be rewritten. For example in line 125 the results start with Fig2A. The authors need to look at some published manuscripts and write the results accordingly.

Author response: We agree with the reviewer’s assessment. We understand the importance of a clear structure in the results section. We have reviewed published manuscripts in this journal to guide our revision and have restructured all of the results section accordingly. The section now introduces findings more clearly, such as Fig 2A, have been appropriately contextualized within the text.

Lines 132-145: We selected different species to further investigate the difference of the *CASP14* expression. We found that the *CASP14* gene was most highly expressed in human skin, followed by sheep skin and cattle skin (Fig 2A). To explore potential sex-related differences in *CASP14* expression across sheep tissues, we analyzed gene expression in male and female sheep (Fig 2B). *CASP14* gene expression in skin tissue was elevated in both female and male subjects relative to other tissues. Furthermore, the expression of the *CASP14* gene was examined across different breeds (Fig 2C). Within the SHE group (Carpet, Rambouillet, Tibetan), *CASP14* gene expression was the lowest while higher expression were noted in scurred sheep breeds (Bashibai, Chinese Merino, Hu, Spanish Churra, Tan). These analyses further highlighted the significance of *CASP14* gene expression in skin and scurred phenotype.

4. The figures need to be labeled properly especially Fig5B.

Author response: We have thoroughly reviewed and adjusted all figure labels to ensure clarity and accuracy, with particular attention to Fig 5B. In Figure 5B, we plotted each locus based on its Fst value, categorizing the loci into four main types: Downstream, Upstream, Exon and No difference. With some locis randomly selected for labeling in the figure.

Figure 5:

5. In their conclusion the authors state that there is a correlation between the CASP14 gene and the size of sheep horns but they never explained in the beginning of the manuscript which sheep has what horn size.

Author response: Thank you for your suggestion. In the Materials and Methods section, we specified that the 15 sheep were divided into two groups: the scurred group, with horn lengths ranging from 0-12 cm, and the SHE group, with horn lengths greater than 12 cm. Detailed horn length for each sheep have also been added to Supplementary Material 1.

Lines 63-67: We collected totaling fifteen Tibetan sheep samples from Dangxiong, Tibet, China. All individuals in this study are female, with ages ranging from 2 to 4.5 years (PRJNA1003277) [18]. The Tibetan sheep were categorized into two groups based on horn characteristics: one with 7 sheep having scurred horns (0–12 cm), and another with 8 sheep with SHE horns (>12 cm). From the 15 sheep, we collected tissue with soft horns, placed them in deep cryopreservation tubes, and stored the tubes in liquid nitrogen.

**Reviewer #2:**

1. Grammar and Spelling

Line 31: "eco-nomic" should be corrected to "economic."

Line 40: "epi-dermis" should be "epidermis."

Line 70: "alongside laboratory-collected samples" is slightly unclear. It would be clearer as "in addition to laboratory-collected samples."

Line 110: "Kompetitive Allele-Specific PCR" should consistently be abbreviated as "KASP" for better flow, as the acronym is introduced later.

Line 221: "coinciding with the formation of the epidermal stratum corneum" should be rephrased for clarity, as the word "coinciding" is somewhat repetitive throughout the article.

Author response: Thank you for your careful review and helpful comments regarding grammar and spelling. We have addressed each point as follows:

We have corrected “eco-nomic” to “economic” on line 31, and we have updated “epi-dermis” to “epidermis” on line 41.

In lines 70-73 of the manuscript, we have rephrased the description to improved clarity.

Line 70-73: A total of 2,915 high-quality RNA-Seq datasets were accessed from the National Center for Biotechnology Information (NCBI) and the European Bioinformatics Institute (EBI), and combined with RNA-Seq data of our 15 sheep for the following procedures.

We have standardized “Kompetitive Allele-Specific PCR” to “KASP” and introduced the abbreviation upon its first mention in lines 113-114.

Lines 113-114: Finally, the identified loci were sent to a biotechnology company for validation through KASP (Kompetitive Allele-Specific PCR) assays.

We have revised the phrase on line 245 of the manuscript to reduce repetition, rephrasing it to improve clarity. The updated wording now reads: “*CASP14* is involved in the formation of the embryonic skin barrier, with its expression initiating from embryonic day 14.5 or 15.5 and processing commencing from day 17.5, occurring alongside with the formation of the epidermal stratum corneum.”

2. Technical Consistency:

"disruption of RXFP2 is associated with testicular descent" lacks context regarding its relevance to horn growth. A clearer link should be made to explain why this finding is significant for the current study.

"low or even undetectable in the blood, CNS, heart, liver, lung and muscle" – the term "CNS" should be spelled out fully the first time it is used, as it may not be immediately recognized by all readers.

Author response: As suggested by the reviewer, we have made some adjustments to our original description to clarify our findings in lines 210-216. In addition, our study highlighted that knocking out *RXFP2*, a gene closely linked to horn growth, did not affect horn size, suggesting that the regulatory mechanisms of horn growth in sheep remain complex and not yet fully understood. This underscores the importance of our study in exploring the gene regulatory mechanisms underlying horn growth, which we aim to investigate further.

lines 210-216: For the first time, researchers utilized CRISPR/Cas9 technology to successfully create a cryptorchid sheep model by targeting the *RXFP2* gene. However, the findings of this study indicated that partial disruption of the *RXFP2* gene did not affect the horn size in sheep, suggesting that partial disruption of *RXFP2* was associated with testicular descent rather than horn formation [33]. This indicated that the gene regulating the sheep horn phenotype was not singular, as demonstrated by the *RXFP2* disruption not affecting horn size, thus necessitating a more in-depth investigation into the genetic mechanisms underlying the sheep horn phenotype.

On line 124: The term “CNS” is now spelled out as “central nervous system” upon its first mention to ensure clarity for all readers.

Line 124: Additionally, The *CASP14* gene exhibited relatively high expression in the skin, while its expression levels were low or even undetectable in the blood, central nervous system (CNS), heart, liver, lung and muscle.

3. Flow and Clarity:

The transitions between sections discussing CASP14's role in apoptosis and its relation to horn growth could benefit from smoother transitions to connect these ideas more explicitly.

More explanation is needed when introducing the Fst analysis results. The significance of Fst values should be briefly clarified for readers unfamiliar with population genetics.

Addressing these minor grammatical and technical inconsistencies would enhance the overall clarity and readability of the article while maintaining the strength of its scientific findings.

Author response: We agree with the reviewer’s assessments. We inserted the explanation on discussing the role of *CASP14* in apoptosis and its relation to horn growth on line 240.

Line 240: The up-regulation of *CASP14* by these lipids is mediated through the inhibition of kinase pathways, highlighting its role in cell differentiation and apoptosis [43]. Which may also impact the development of the horn phenotype.

In lines 103-105 of the manuscript, we have added a brief explanation of Fst value at its first mention to help clarify their significance for readers.

Lines 103-105: F-statistic (Fst) values were calculated using VCFtools (v.0.1.16) to identify SNP loci exhibiting significant differences between these two populations. Fst value is a measure of genetic differentiation between populations, with higher values indicating greater genetic divergence.

We have addressed these minor issues to improve the article's clarity and readability while preserving the strength of our findings.
